# Lipidomics of polarized macrophages in the human adipose tissue

**DOI:** 10.1038/s41598-025-32912-z

**Published:** 2025-12-19

**Authors:** Vladimír Vrkoslav, Kateřina Pražáková, Štěpán Strnad, Karel Paukner, Barbora Muffová, Soňa Kauerová, Jiří Froněk, David Sýkora, Josef Cvačka, Rudolf Poledne, Marek Petráš, Ivana Králová Lesná

**Affiliations:** 1 Institute of Organic Chemistry and Biochemistry, Czech Academy of Sciences, Prague, Czech Republic; 2 Department of Analytical Chemistry, University of Chemistry and Technology Prague, Prague, Czech Republic; 3 Institute for Clinical and Experimental Medicine, Prague, Czech Republic; 4 Department of Physiology, Faculty of Science, Charles University in Prague, Prague, Czech Republic; 5 Department of Analytical Chemistry, Faculty of Science, Charles University in Prague, Prague, Czech Republic; 6 Department of Epidemiology and Biostatistics, 3 Faculty of Medicine, Charles University, Prague, Czech Republic; 7 Department of Anesthesiology, Resuscitation and Intensive Care Medicine, 1st Faculty of Medicine, Charles University and Military University Hospital, Prague, Czech Republic

**Keywords:** Adipose tissue, Ether phospholipids, Inflammation, Lipids, Obesity, Phospholipids, Pro-inflammatory macrophages, Biochemistry, Diseases, Immunology

## Abstract

Macrophages are critical components of the innate immune system that adapt their function based on local microenvironmental cues. In adipose tissue, macrophage polarization is intimately linked to metabolic regulation and inflammation. In this pilot study, we performed lipidomic profiling to characterize the phospholipid composition of pro-inflammatory (PI-ATM) and non-inflammatory (NI-ATM) macrophages isolated from visceral adipose tissue of living kidney donors. Tissue samples were processed, and macrophages were isolated and sorted using CD14 and CD16 markers. Lipids were extracted by methyl *tert*-butyl ether, and differences in the lipidome were analyzed using the optimized UHPLC-MS method. PI-ATM exhibited a higher abundance of lipids across various lipid classes than NI-ATM. Phosphatidylethanolamines and ether lipids derived from phosphatidylethanolamines and phosphatidylcholines were most frequently found among the significantly altered lipids. The majority of significantly altered lipid species contained an octadecenoic aliphatic chain. These findings suggest that alterations in membrane lipid composition - particularly ether lipids and phosphatidylethanolamines - may play a pivotal role in macrophage polarization and adipose tissue inflammation. Our study provides novel insights into the lipidomic regulation of macrophage function in human adipose tissue and identifies potential targets for therapeutic intervention in metabolic disorders.

**Supplementary Information:**

The online version contains supplementary material available at 10.1038/s41598-025-32912-z.

## Introduction

Macrophages are highly versatile cells of the innate immune system that can adapt their functionality based on their microenvironment, enabling them to perform specialized roles even under normal physiological conditions. These immunocytes eliminate infectious organisms and play a crucial role in maintaining tissue and organismal homeostasis^1^. Dynamic changes in the microenvironment can drive specific alterations in macrophage behavior, allowing them to adjust immune cell functioning appropriately.

 In vitro studies have explored both pro-inflammatory and non-inflammatory macrophage phenotypes (PI-ATM and NI-ATM), identifying the stimulatory factors and characterizing the associated phenotypic and functional changes for each. However, a major limitation of this approach is its inability to imitate the complex microenvironment that macrophages encounter in specific tissues. For example, macrophages in adipose tissue are exposed to unique conditions, including high levels of fatty acids, insulin, metabolites, and adipose-derived hormones, which significantly influence their behavior. Overfeeding induces metabolic dysregulation, leading to the activation and polarization of inflammatory macrophages in adipose tissue. A strong connection between metabolic dysregulation in adipose tissue and the liver has been observed^2^. Resident macrophages in these tissues buffer fluctuation in intercellular lipids and maintain tissue homeostasis^3,4^. We were able to document that polarization changes occur even in the adipose tissue of healthy individuals, and these changes are related to factors of cardiovascular disease (body mass index (BMI), age, menopause, and non-HDL cholesterol)^5–7^. The specific phenotypes of metabolically activated PI-ATM, which, in addition to CD16, also strongly express CD36, characterize adipose tissue dysfunction. Our published data further demonstrated a direct positive link between PI-ATM and adipose tissue membrane lipids, namely palmitate and palmitoleate. In contrast, an opposite link was shown for omega-3 fatty acids^8^.

Over the years, numerous interactions between lipids and inflammation have been identified and extensively studied. Lipids influence membrane fluidity, which is closely connected with essential macrophage functions^9,10^. This link between inflammation and lipid metabolism was also shown in intracellular cell membranes, namely endoplasmic reticulum membranes^11^, mitochondrial, lysosomal, and endosomal macrophage membranes. These data suggest that changes in the lipidome act as modulators of inflammation. Our earlier work^8^ also demonstrated a pro-inflammatory effect of saturated fatty acids and, conversely, an anti-inflammatory effect of monounsaturated and polyunsaturated fatty acids. The pilot data we present here clearly identify the effect of lipid species on the polarization of pro-inflammatory macrophages. However, mutual effects cannot be excluded, as in vitro experiments revealed differences in the utilization of exogenous fatty acids between PI-ATM and NI-ATM^12^. Also, another study demonstrated that pro-inflammatory stimuli per se can alter macrophage lipidome^13^ or suppress lipid metabolic pathways. In conclusion, cellular lipid metabolism has emerged as an integral part of maintaining macrophage regulation and function as an integral part of the immune system homeostasis. Specifically, exposure of macrophages to phosphatidylcholine (PC) or phosphatidylserine (PS) triggers macrophage activity^14^.

Analyzing our data regarding the link between polar lipids and macrophage polarization in the context of the above-mentioned data leads us to focus on a more detailed lipidomic analysis of ATM’s phospholipid structure.

## Methods

### Characterization of individuals enrolled, sample preparation, and cell sorting

Living kidney donors were enrolled in the study performed at the Institute for Clinical and Experimental Medicine in Prague, Czech Republic. All were fully informed about the process of kidney donation and transplantation, as well as adipose tissue sampling during organ cleaning before transplantation. All individuals signed informed consent forms. The study’s design complied with the ethical principles of the Declaration of Helsinki and was approved by the Ethics Committee of the Institute for Clinical and Experimental Medicine and Thomayer Hospital, Prague, Czech Republic (No. 31113/23).

Lipoprotein concentrations and BMI of living kidney donors (LKD) were compared to the representative population sample. Thirty age and sex-matched individuals were selected from the participants of the WHO PostMONICA study, in which cardiovascular risk factors of 1% sample of 25-65-year-old individuals in 9 regions of the Czech Republic were analyzed (*n* = 2750).

**Fig. 1:**
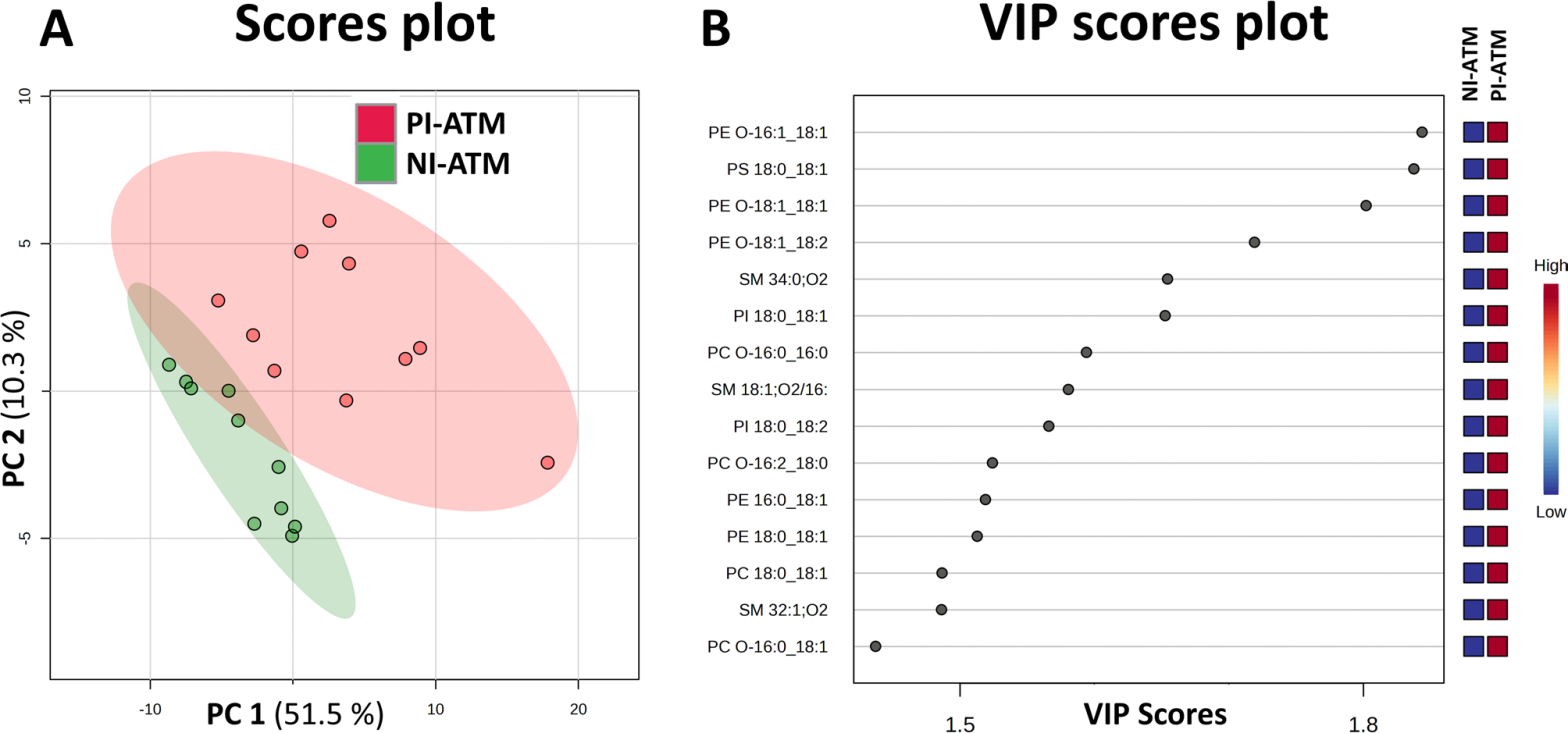
PLS-DA of lipid profiling of pro-inflammatory and non-inflammatory adipose tissue macrophages. (**A**) Least squares discriminant analysis (PLS-DA) score plot based on lipid profiling of pro-inflammatory and non-inflammatory adipose tissue macrophages (PI-ATM and NI-ATM, respectively). (**B**) variable importance in projection (VIP) score plot showing the top 15 (score > 1.4) most important lipid features identified by PLS-DA. Colored boxes (on right) indicate relative concentration of corresponding metabolite.

For the final experiment, perirenal visceral adipose tissues were obtained from 10 living kidney donors (enrolled between March 2023 and March 2024) and processed immediately during hand-assisted nephrectomy. The samples were cleaned of all visible blood vessels and fibrous tissue and then finely minced by scissors. Following the wash-out residual blood, the adipose tissue was enzymatically digested in a collagenase solution (2 mg/ml; Collagenase from *Clostridium histolyticum*, CAS No. 9001-12-1, Sigma Type II, Sigma-Aldrich) prepared in 2% bovine serum albumin (BSA) in Dulbecco’s Phosphate-Buffered Saline (DPBS) without calcium and magnesium. Digestion was conducted in a shaking water bath at 37 °C. The resulting cell suspension was promptly cooled on ice, filtered through a 150 μm and 50 μm strainer, washed with DPBS containing BSA, and centrifuged. The cell suspension was subsequently labeled with fluorescently conjugated antibodies, including CD14-FITC (FITC Mouse Anti-Human CD14, BD Biosciences) and CD16-PE (Anti-Hu CD16 PE, EXBIO). Labeling was performed for 30 min at room temperature in the dark. Following antibody incubation, the cells were washed, and the pellet was resuspended in Hanks’ Balanced Salt Solution. 7-Aminoactinomycin D (7-AAD, BD Biosciences) was added to the cell suspension to assess cell viability. The prepared sample was then subjected to cell sorting using the BD FACSMelody system to isolate viable CD14⁺CD16⁺ and CD14⁺CD16⁻ cell populations. Gating strategy is shown in Sup. I. Figure 1. In this study, macrophages were isolated from visceral adipose tissue and sorted based on CD14 and CD16 expression, with CD14⁺CD16⁺ cells representing a pro-inflammatory subset (PI-ATM) and CD14⁺CD16⁻ cells representing a non-inflammatory phenotype (NI-ATM). On average we yielded 40,917 PI-ATM cells (min. 14859- max. 93671 cells/sample) and 42,169 NI-ATM cells (min. 11706- max. 78108 cells/sample) per sample. Isolated and sorted cells were stored in -80 °C until UHPLC-MS analysis. This simplified phenotyping is consistent with our previous studies on human ATMs^5,15^, and was used to enable sufficient cell yield for untargeted lipidomic analysis from small human tissue samples, balancing precise phenotypization with feasibility.

## UHPLC-MS analysis

Solvents and additives of LC-MS purity were used for sample preparation and UHPLC-MS analysis: acetonitrile (VWR, PA, USA), water (Fisher Chemical, MA, USA), 2-propanol (Merck, NJ, USA), formic acid (Thermo Scientific, MA, USA), ammonium formate (Honeywell, NC, USA), and methanol (VWR, PA, USA). Lipids were extracted from macrophages using the methyl *tert*-butyl ether (MTBE, for HPLC, Honeywell, NC, USA) extraction^16^ with some modifications. Due to limited donor material and uncertain cell yields, we used the maximum number of cells available for each sample. Briefly, samples were placed in 2 mL Eppendorf tubes with 150 µL of cold methanol. 10 µL of EquiSPLASH (Avanti Polar Lipids, AL, USA) was added, and samples were sonicated for 10 min. Then 500 µL of cold MTBE was added, and the mixture was mixed at room temperature (1 h). In the next step, 125 µL of water was added, the mixture was mixed (5 min), and centrifuged (10 min, 500 g). The volume of 400 µL of the upper phase was transferred to a separate vial. The lower phase was re-extracted with 200 µL of the solvent mixture MTBE/methanol/water (10:3:2.5, v/v/v). The extraction was repeated, and 200 µL of the upper phase was combined with the first upper phase. The phases were evaporated and reconstituted in 200 µL of methanol/2-propanol (1:1, v/v). Untargeted lipidomics profiling was performed by coupling a Vanquish UHPLC chromatography system with TriPlus RTC autosampler to an Orbitrap IQ-X Tribrid mass spectrometer (ThermoFisher Scientific, MA, USA) with heated electrospray ionization. Mobile phase A was acetonitrile/water (60:40 (v: v)), and mobile phase B was 2-propanol/acetonitrile (90:10 (v: v)). Both mobile phase solutions contained 10 mM ammonium formate and 0.1% formic acid. The separations were performed on the Waters Acquity UPLC BEH C18 (2.1 × 100 mm, 1.7 μm) column operated at 55 °C and at a flow rate of 300 µL/min, with the elution gradient program as follows: 0–2.0 min, 0–43% B; 2.0–2.1 min, 43–55% B; 2.1–12.0 min, 55–65% B; 12.0–18.0 min, 65–85% B; 18.0–20.0 min, 85–100% B; 20.0–25.0 min, 100% B; 25.0–25.1 min, 100–0% B; and 25.1–30.0 min, 0% B. The injection volume was 5 µL for positive and 10 µL for negative ion modes. Full scan and MS^2^ HCD data acquisition were performed in a data-dependent setting for both ionization modes separately in the range of *m/z* 250–1600, 1 s cycle time, dynamic exclusion after 1 time for 3 s. The MS resolution 120 000 and the MS/MS resolution 15 000 were applied. The electrospray ionization voltage and the transfer capillary temperature were set to 3.5 kV and 350 °C, respectively. Quality control samples were prepared by pooling equal aliquots from all the samples in the study. These pooled QC samples were injected at the beginning, middle, and end of LC-MS sequence to monitor instrument stability and reproducibility. Lipid identification was performed using MS-DIAL 5^17^. The following parameters were set in MS-DIAL: MS1 0.01 Da, MS2 0.025 Da, minimum peak height 10,000 amplitude. QC samples were used as the reference files for sample alignment. Features with 3-fold change sample max/blank average were filtered. Only the referenced matched annotations with match score > 1.3 were kept. Lipid annotations in MS-dial were manually cross-checked to ensure consistency between retention behavior and the degree of unsaturation to remove false positive identifications. Only the lipid annotation specifying the number of carbon atoms in each individual aliphatic chain and the number of double bonds were accepted, except for sphingomyelins (SM). Furthermore, annotated lipids exhibiting high abundance in blank samples were excluded from further consideration. To maximize detection sensitivity and minimize background lipid contamination, we performed extensive optimization of the HPLC-MS method, including solvent system testing based on the approach published by Čajka et al.^18^. Normalization to cell count was performed during data processing.

### Statistical analysis

The power of the test for altered lipid levels in the CD16⁺ subset relative to the CD16⁻ subset was estimated using a paired two-sample means test, with means calculated from log-transformed lipid concentrations. The null hypothesis assumed a difference in lipid concentrations ≤ 0.176 (corresponding to a concentration ratio of 1.5). The alternative hypothesis was accepted when the observed difference including lower limit of 95% confidence interval surpassed this threshold. A sample size was considered sufficient if the statistical power exceeded 80% (0.8). Other statistical analysis was done with MetaboAnalyst software 6.0 (http://www.metaboanalyst.ca/). Before statistical analysis, the data were subjected to auto scaling. Volcano plot analysis was used to identify altered lipids (fold change > 2 and p-value < 0.05 by Student’s paired t-test). During processing, all “n.d.” values are replaced with one-fifth of the minimum positive value of the feature.

## Results

Data of age, BMI, and lipoprotein parameters of the analyzed group of LKD did not differ in main clinical characteristics from the representative population sample of selected individuals (sex and age-matched), Table 1. This documents that the group of LKD was very similar to the general population. Only two of the LKD have a BMI of more than 30 kg/m^2^ (with a variation within group 22.31–30.78). The mean BMI in this group (27.65 ± 2.8 kg/m^2^) agrees with the recent shift in body composition and increase of visceral adipose tissue in most of the world’s populations^19^.

**Table 1:** Characteristics of the group of living kidney donors compared to the Czech population sample (PostMONICA study, *n* = 30), age and sex-matched sample of 2750 individuals. n.s. - nonsignificant.

	Living kidney donors	Population sample	Significance
Age (years)	47.95	47.97	n.s.
Total cholesterol (mmol/l)	5.52	5.26	n.s.
HDL cholesterol (mmol/l)	1.52	1.59	n.s.
LDL cholesterol (mmol/l)	3.51	3.13	n.s.
Triglycerides (mmol/l)	1.07	1.14	n.s.
BMI (kg/m^2^)	27.65	27.73	n.s.

Based on untargeted lipidomic profiling of macrophages isolated from human adipose tissue, a total of 96 annotated lipid species were used for subsequent statistical analysis and comparative evaluation. In addition, over 90 triacylglycerol (TAG) species were annotated. However, they were excluded from statistical evaluation due to the inherently high abundance of TAGs in adipocytes and the possibility of nonspecific adsorption to macrophage surfaces, which could bias the interpretation.

Due to the limited amount of biological material, we were able to isolate only tens of thousands of macrophages per sample, representing approximately a tenfold reduction compared to the typically recommended minimum of 500,000 cells for robust lipidomic analysis^20^. This limitation significantly constrained the lipid detection and contributed to the relatively low number of reliably annotated lipid species. Nevertheless, significant differences in the lipidome were observed between NI-ATM and PI-ATM.

Among the annotated compounds, several ether lipids were identified and most likely correspond to plasmalogens^21^. However, the low abundance of these minor species limited spectral quality. At such low concentrations, diagnostic ions required for unambiguous identification of (1Z)-alkenyl ether linkages were not observed in HCD MS/MS spectra. Although statistically significant differences were observed for ether lipids, their low absolute concentrations and the untargeted nature of the lipidomic analysis precluded definitive discrimination between plasmalogens and other ether lipid forms.

**Fig. 2:**
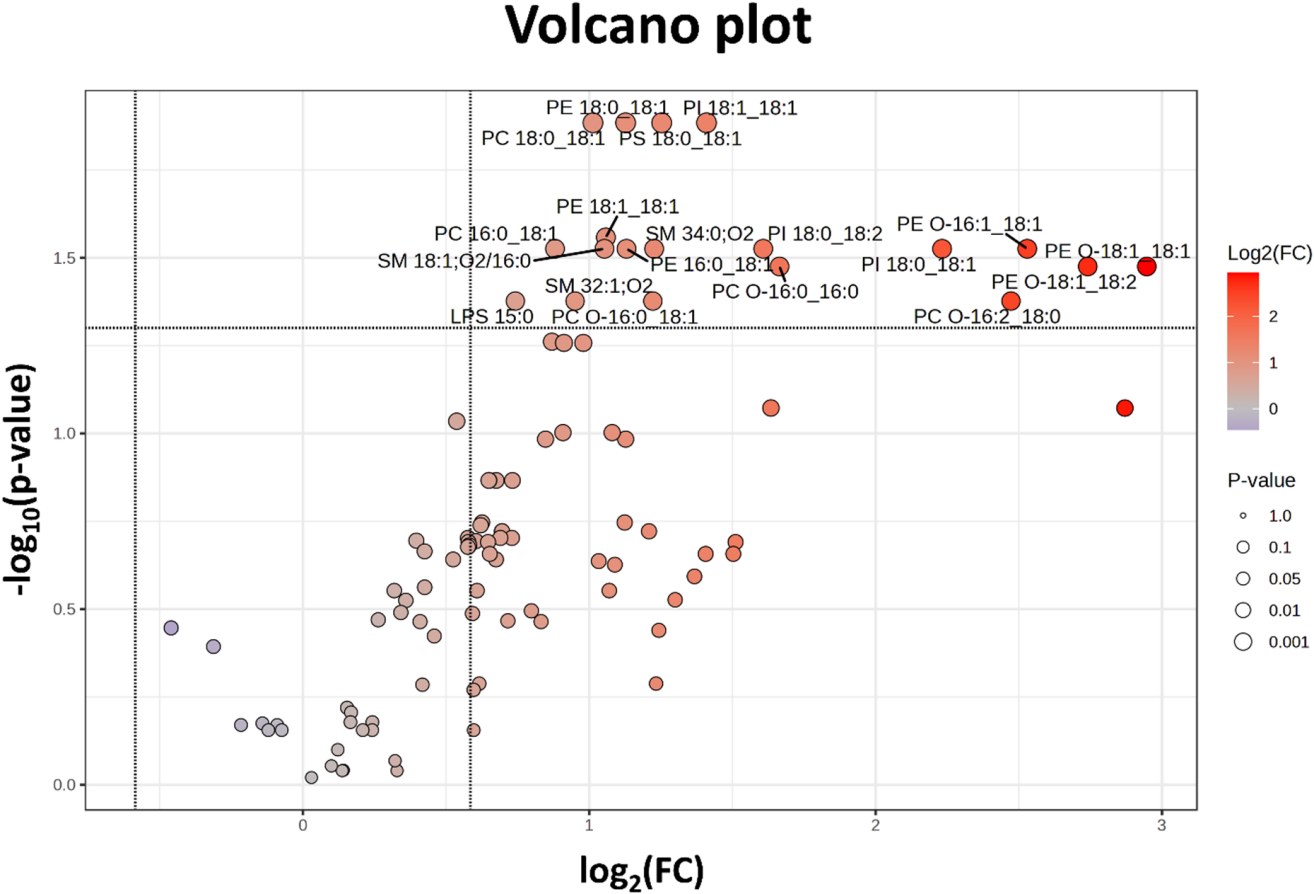
Volcano plot of intact lipids. Volcano plot of lipids indicating lipid species that are significantly increased in the pro-inflammatory adipose tissue macrophages (PI-ATM) compared with non-inflammatory adipose tissue macrophages (NI-ATM) (fold change (FC) > 2 and *p*-value < 0.05 by Student’s paired t-test).

**Fig. 3:**
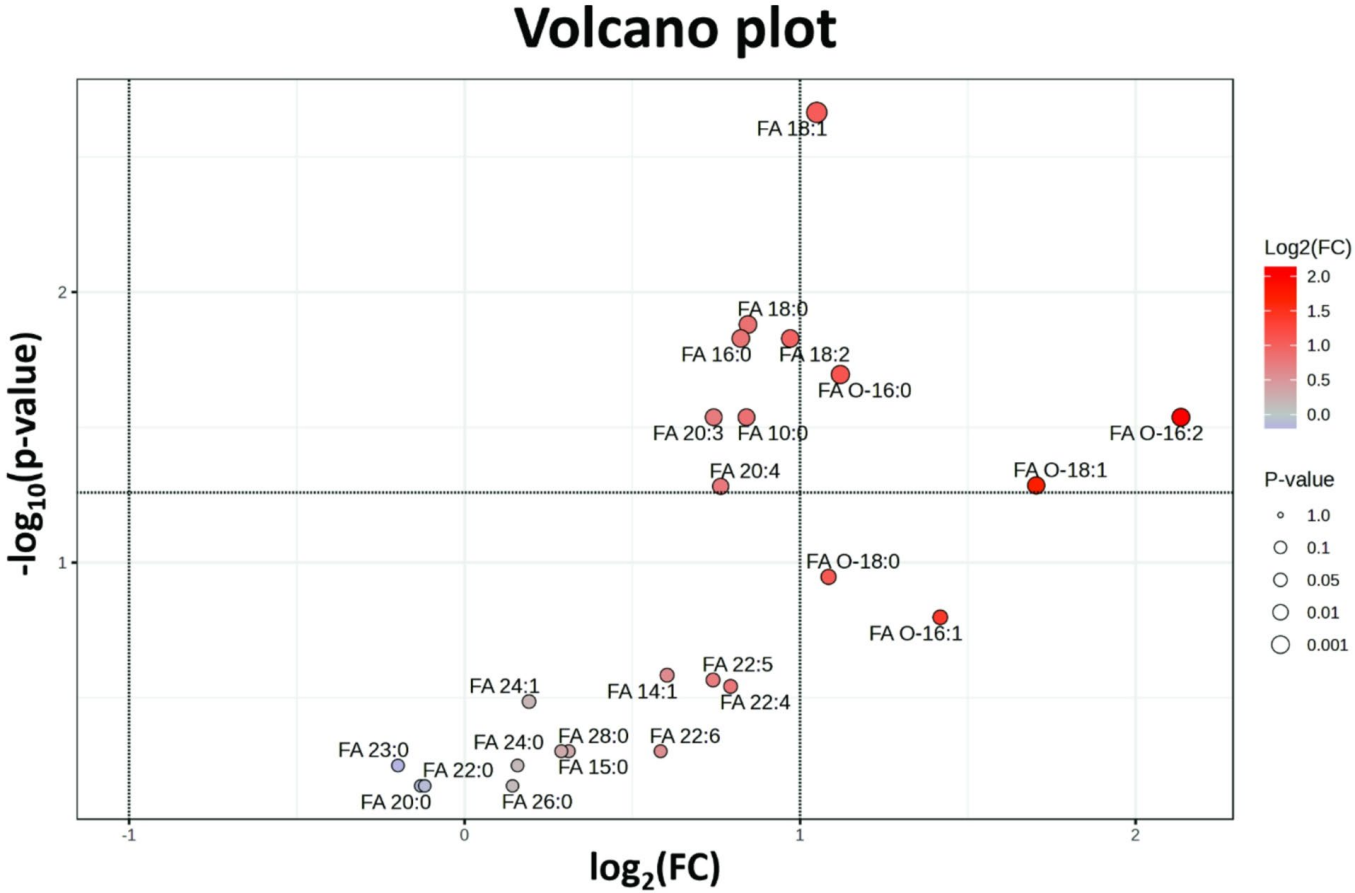
Volcano plot of ester-linked and ether-linked aliphatic chains. Volcano plot of ester-linked and ether-linked aliphatic chains indicating aliphatic moieties that are significantly increased in the pro-inflammatory adipose tissue macrophages (PI-ATM) compared with non-inflammatory macrophages (NI-ATM) (fold change (FC) > 2 and *p*-value < 0.05 by Student’s paired t-test).

The analysis indicates that PI-ATM contained a higher abundance of lipids across multiple lipid classes than NI-ATM (Sup. II Table 1). The lipidomic profiles were analyzed using partial least squares discriminant analysis (PLS-DA), a supervised multivariate method that identifies linear combinations of variables to optimally separate predefined groups. PLS-DA analysis revealed a clear separation between PI-ATM and NI-ATM macrophages (Fig. 1: A; cross-validation is shown in Sup. I. Figure 2). Notably, NI-ATM exhibited tighter clustering, indicating greater homogeneity among their lipid profiles compared to the more dispersed clustering observed for PI-ATM. A variable importance in projection (VIP) score plot, a key output of PLS-DA, ranked lipid features based on their contribution to group separation (Fig. 1: B). Higher VIP scores indicated lipids that play a significant role in distinguishing between groups. The analysis revealed that ether lipids (PE O- and PC O-) constitute 4% of the 15 most important lipids, indicating their significant role in differentiating the studied groups. Furthermore, most of the significant lipid features contained an 18:1 aliphatic chain, suggesting a potential structural or functional relevance of this fatty acid moiety in the observed lipidomic variations. A paired t-test offered an additional perspective on the results. As a parametric test, it compares the means of two related (paired) samples to determine whether there is a significant difference in lipid levels between them. The results are presented as a heatmap (Sup. I. Figure 3) highlighting 15 lipid species with a p-value < 0.05. The color coding reflects the relative abundance of lipid species in PI-ATM and NI-ATM macrophages. The paired t-test results aligned with the PLS-DA analysis, highlighting again plasmalogens as prominent among the significant lipid features, along with many lipids containing an 18:1 chain. Notably, 0% of the annotated lipid features identified in the paired t-test also overlap with those ranked by PLS-DA VIP scores, reinforcing the robustness of the findings.

Another statistical approach, the Volcano plot with FDR correction, was employed to identify significantly altered lipid features. In this plot, the x-axis represents the log_2_-transformed fold change (FC) of lipid species between paired conditions, while the y-axis displays the statistical significance as − log_10_​(p-value). Lipid features with large fold changes and high statistical significance appear in the top left (downregulated) and top right (upregulated) regions of the plot. A Volcano plot analysis, using a significance threshold of *p* < 0.05 (Student’s paired t-test) and an FC > 2, identified 19 upregulated lipids in PI-ATM compared to NI-ATM (Fig. 2, Sup I. Table 1). To evaluate whether the sample size used in the pilot study was sufficient, a post hoc statistical power analysis was performed. The analysis was based on the concentration of significantly altered lipid species identified via volcano plot analysis. Using these values, the statistical power of the test was estimated for each of the 19 analytes (Sup I. Table 1). For 4 out of the 19 analytes, the estimated power exceeded the conventional threshold of 80%. Based on these calculations, the sample size employed in the pilot study can be considered adequate for drawing preliminary conclusions. According volcano plot analysis, phospholipids, including phosphatidylcholines (PC, ether-linked PC O-), phosphatidylethanolamines (PE, ether-linked PE O-), phosphatidylserine (PS), sphingomyelins (SM), and phosphatidylinositol (PI), showed the most pronounced relative increases. These lipids were predominantly enriched in saturated stearic acid (18:0) and monounsaturated oleic acid (18:1). Consistent with previous statistical analyses, ether-linked PC and PE exhibited the highest fold changes and were among the most strongly upregulated lipids (right side of Fig. 2). PC, PS, PE, and PI species containing fatty acid (FA) 18:1 or a combination of FA 18:1 and 18:0 exhibited the highest − log_10_ (*p*-value), as shown in Fig. 2 (top center).

In the final statistical analysis, we examined the macrophage lipidome at the level of ester- and ether-linked aliphatic chains. To achieve this, total concentrations of individual aliphatic chains were extracted from the lipidomic data and compared using the Volcano plot with FDR correction. The plot (Fig. 3) revealed a highly significant increase in the content of monounsaturated fatty acid FA 18:1. Furthermore, a significant increase was also observed for ether-linked aliphatic chains (O-16:0, O-18:1, and O-16:2) in PI-ATM.

In summary, statistical analyses revealed significant alterations in lipid profiles between PI-ATM and NI-ATM. Phospholipids, particularly plasmalogens, were crucial in distinguishing between these groups. In PI-ATM, multiple phospholipid classes were upregulated compared to NI-ATM, including PC, PC O-, PE, PE O-, PS, SM, and PI. At the fatty acyl chain level, in PI-ATM, a highly significant increase was observed in monounsaturated FA 18:1 and ether-linked aliphatic chains, including O-16:0, O-18:1, and O-16:2.

## Discussion

Our findings reveal distinct untargeted lipidomic profiles between CD14⁺CD16⁺ and CD14⁺CD16⁻ macrophages in human visceral adipose tissue. The enrichment of phosphatidylethanolamines and ether-linked phospholipids in CD14⁺CD16⁺ macrophages is associated with their pro-inflammatory characteristics. While it remains unclear whether lipid composition drives macrophage polarization or results from it, the association suggests a functional link between membrane lipids and inflammatory status. These insights highlight the relevance of lipidomic profiling in understanding adipose tissue inflammation and its potential in identifying therapeutic targets.

In this study, we simplified the identification of adipose tissue macrophage (ATM) subpopulations using CD16 as the sole discriminatory marker. Due to limitations in sample volume and cell yield from living donor biopsies, we adopted this streamlined panel to ensure sufficient cell numbers for high-resolution lipidomic analysis. Notably, our previous work has shown that CD16⁺ ATMs express key pro-inflammatory genes at significantly higher levels than their CD16⁻ counterparts^15^. Furthermore, we previously identified an adipose tissue-specific pro-inflammatory subpopulation—characterized by high CD36 expression—that is exclusively present within the CD16⁺ subset, where it comprises 65–70% of all macrophages^22^. In contrast, nearly all CD16⁻ macrophages express the marker CD163 and display only low levels of CD36. This strategy does not encompass the full phenotypic diversity of ATMs, such as markers like CD206, CD163, and HLA-DR. We consider the use of CD14 and CD16 both appropriate and sufficient, given the study’s aims and the practical constraints of limited sample volume and cell yield. However, based on these findings, along with recent data challenging the specificity of CD163 as a definitive anti-inflammatory marker and the lack of significant differences in anti-inflammatory gene expression between CD16⁺ and CD16⁻ macrophages, we chose to use the broader term ‘non-inflammatory.’ This designation encompasses not only anti-inflammatory macrophages but also a broader spectrum of cells that lack definitive pro-inflammatory markers. 

The age, BMI, and lipoprotein parameters of the LKD group did not differ from the representative population sample selected from the 1% population 25–64 years sample of the WHO PostMONICA survey in years 2016–2017^23^. Therefore, we suppose that the studied subjects are representative of the Czech population in parameters of lipid metabolism. The mean BMI of 27.77 ± 2.77 kg/m^2^ documents that the Czech population is also shifting to more frequent overweight and obesity^19^. Interestingly, two LKD individuals with a BMI over 30 decreased the difference between the lipidomes of the two macrophage subpopulations (Red – PI-ATM, Green – NI-ATM in the heat map, Sup. I, Fig. 2).

Using the PLS-DA method to compare 96 lipid molecules with the lipid profiles of differently polarized macrophages in adipose tissue, we documented a clear separation between the two polarized groups (Fig. 1A). Figure 1B shows the lipids that contribute the most to the differentiation of these groups. The 95% confidence intervals slightly overlap. The confidence interval covers a larger area because there is a greater variation between points in the PI-ATM group. Nevertheless, only one NI-ATM point falls within the confidence interval of PI-ATM. The broader lipid profile observed in PI-ATM may result from our simplified classification of macrophages into two subpopulations. We demonstrated, that these subpopulations exhibit a more heterogenous nature compared to CD16 negative NI-ATM^22^.

Ether phospholipids, most commonly found as plasmalogens, have been largely neglected despite being isolated nearly a century ago and representing about 20% of total cellular phospholipids^24^. Ether lipids are present in various organs and lipoproteins^25^, and their importance is underscored by the steadily increasing number of publications on the topic^26^. They play a substantial role in maintaining membrane fluidity and are critical for molecular signaling^27,28^.

We were able to document the most significant differences in the plasmalogen content between PI-ATM and NI-ATM. Ether-bound fatty acids in the phosphatidyl ethanolamine and phosphatidyl choline are significantly increased in PI-ATM compared to NI-ATM (Fig. 2). We suppose that these lipids play an important role in the different polarization of macrophages in the adipose tissue of the LKD. It agrees with the published data^21,29^. In the both studies authors demonstrated that a decrease in cell fluidity is connected with a decrease in plasmalogens, and following plasmalogens supplementation, it can restore cell membrane fluidity. The PI-ATM function change might represent metabolic changes connected with the pathophysiology of adipose tissue^30^ moving from the lean adipose tissue to the increased volume of visceral tissue.

These data are in agreement with the importance of plasmalogens in pro-inflammatory macrophages playing a substantial role in cancer pathology^31,32^, obesity^33^, Alzheimer’s disease^34^, and even longevity^35^. In situ, polarization of adipose tissue macrophages is probably another pathology in which plasmalogens play a substantial role. It is important to stress that the increase of plasmalogens in PI-ATM in our study was documented in the group of LDK without substantial obesity presence.

Over the past decade, numerous studies have highlighted the critical role of membrane lipids in regulating macrophage function. Lipidomic has reached a groundbreaking advancement, enabling detailed analysis and interpretation of the effects of specific lipid species on human health. A recent lipidomic study^36^ analyzing over 800 lipid species shed light on their roles in transitioning from health to diseases, including those with known immunological backgrounds.

The importance of phosphatidylethanolamine in biological membranes has been recognized for several decades^37^, although its regulatory mechanisms remain unclear. Nevertheless, phosphatidylethanolamine contributes to plasma membrane structure^38^, microsomal membrane composition^37^, inflammation modulation^39^, and hepatic steatosis in experimental models^40^. The differences in phosphatidylethanolamine are the most important differences distinguishing PI-ATM and NI-ATM (Figs. 1B and 2).

One possible explanation of the phosphatidylethanolamine effect has been described in detail in an experimental model of steatohepatitis^41^. The authors demonstrated that replacing fatty acid in position 2 with oleic acid regulates the accumulation of triacylglycerols within hepatocytes (via intracellular receptors) and promotes liver steatosis. We would like to hypothesize that this mechanism might explain lipidomic differences between PI-ATM and NI-ATM, such as phosphatidylethanolamine, with a significant difference in oleic acid content (Figs. 2 and 3).

The wide range of anti-inflammatory effects of oleic acid on immune cells has been recently reviewed^42^. Studies have demonstrated that oleic acid influences pro-inflammatory genes and cytokine production in macrophages^43^. Oleate was shown to protect adipose tissue macrophages from apoptosis induced by saturated fatty acids, primarily through the downregulation of CD36^44^, a receptor that plays a central role in ATM pro-inflammatory polarization. Saturated fatty acids released from adipocytes induce pro-inflammatory changes in macrophages via TLR-4, triggering TNFα production. TNFα, in turn, activates lipolysis in adipocytes, further releasing saturated fatty acids and fueling a self-sustaining paracrine loop^45^.

Since this study is observational, it does not establish causality. Nonetheless, our findings are consistent with in vitro studies that have actively modulated macrophage polarization and plasma membrane composition. These studies involved either transformed macrophage-like cells^46^ or monocytes isolated in vivo - from animal models^47^ or human blood donors^21^ - that were subsequently polarized under controlled conditions. Notably, the only published study to directly influence myeloid cells in vivo, conducted in genetically modified zebrafish, demonstrated that plasmalogen metabolism is crucial for immune cell activation and modulation^48^. Although our observations are hypothesis-generating, they complement existing data and offer deeper insights into the role of plasmalogens in the immune system. An unusual preferential ether binding of palmitate and palmitoleate^8^ to the surface phospholipids of pro-inflammatory macrophages might direct further research of the macrophage role in the pro-inflammatory status of the adipose tissue and possible general pro-inflammatory shift.

## Conclusion

In conclusion, our data adds significant new knowledge concerning how phospholipid composition relates to macrophage polarization in the adipose tissue. The lipid profile clearly separates PI-ATM and NI-ATM (Fig. 1). Data presented here indicate that ether lipids play a more significant role in adipose tissue homeostasis and inflammation. In addition, phosphatidylethanolamine might play a significant role in the difference between the two types of macrophages in adipose tissue. Although the mechanism of the regulative role of phosphatidylethanolamine as well as plasmalogens is still veiled, our data might stimulate following research in the field of macrophage polarization in adipose tissue.

## Supplementary Information

Below is the link to the electronic supplementary material.

Supplementary Material 1

Supplementary Material 2

## Data Availability

The mass spectrometry data have been deposited in the public repository Zenodo under [https://doi.org/10.5281/zenodo.15296922](https:/doi.org/10.5281/zenodo.15296922).

## Abbreviations

7-AAD: 7-Aminoactinomycin D
ATM: Adipose tissue macrophages
NI-ATM: Non-inflammatory adipose tissue macrophages
BSA: Bovine serum albumin
PC O-: Ether-linked phosphatidylcholine
FA: Fatty acid
HCD: Higher-energy collisional dissociation
PLS-DA: Least squares discriminant analysis
LKD: Living kidney donors
BMI: Body mass index
MTBE: Methyl tert-butyl ether
PC: Phosphatidylcholine
PE: Phosphatidylethanolamines
PE O-: Phosphatidylethanolamines ether-linked
PI: Phosphatidylinositol
PS: Phosphatidylserine
PI-ATM: Pro-inflammatory adipose tissue macrophages
VIP: Variable importance in projection
