# Supplementary material for: Lipidomics of polarized macrophages in the human adipose tissue
Source: Sci Rep. 2025 Dec 19;16:3018. doi: 10.1038/s41598-025-32912-z (PMC12827255; doi:10.1038/s41598-025-32912-z)
Supplement: Supplementary file 1 — Supplementary Material 1 [file 41598_2025_32912_MOESM1_ESM.pdf]

Supplemental data I.

Lipidomics of polarized macrophages in the human adipose tissue

Vladimír Vrkoslav[a], Kateřina Pražáková[a,b], Štěpán Strnad[a], Karel Paukner [c,d], Barbora Muffová[c,d], Soňa Kauerová[c], Jiří Froněk[c], David Sýkora[b], Josef Cvačka[a,e], Rudolf Poledne[c], Marek Petráš[g], Ivana Králová Lesná[c,f*]

[a] *Institute of Organic Chemistry and Biochemistry of the Czech Academy of Sciences, Prague, Czech Republic*

[b] *Department of Analytical Chemistry, University of Chemistry and Technology Prague, Prague, Czech Republic*

[c] *Institute for Clinical and Experimental Medicine, Prague, Czech Republic*

[d] *Department of Physiology, Faculty of Science, Charles University in Prague, Prague, Czech Republic*

[e] *Department of Analytical Chemistry, Faculty of Science, Charles University in Prague, Prague, Czech Republic*

[f] *Department of Anesthesiology, Resuscitation and Intensive Care Medicine, 1st Faculty of Medicine, Charles University and Military University Hospital, Czech Republic*

[g] *Department of Epidemiology and Biostatistics, Charles University in Prague-Third Faculty of Medicine Charles University, 100 00 Prague, Czech Republic*

* Corresponding authors: ivka@ikem.cz

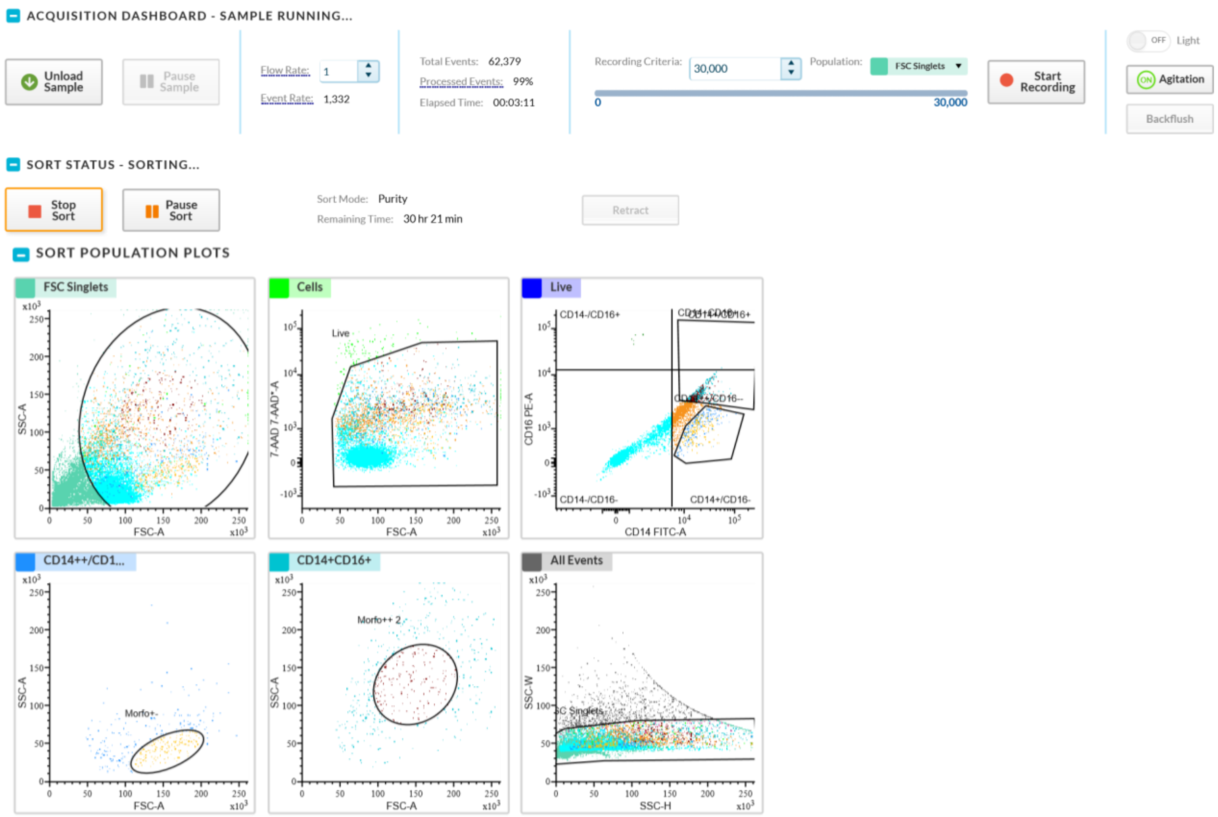

Sup. I Fig. 1: **Gating strategy in cell sorter**. Debris, non-singlets, and dead cells were excluded. Subsequently, CD14^{+}CD16^{+} and CD14^{+}CD16^{-} cell populations were gated. Cells were further selected based on characteristic morphology using side scatter (SSC) and forward scatter (FSC) parameters to minimize contamination by non-specific cell types. The final sorted populations included CD14^{+}CD16^{+} and CD14^{+}CD16^{-} cells.

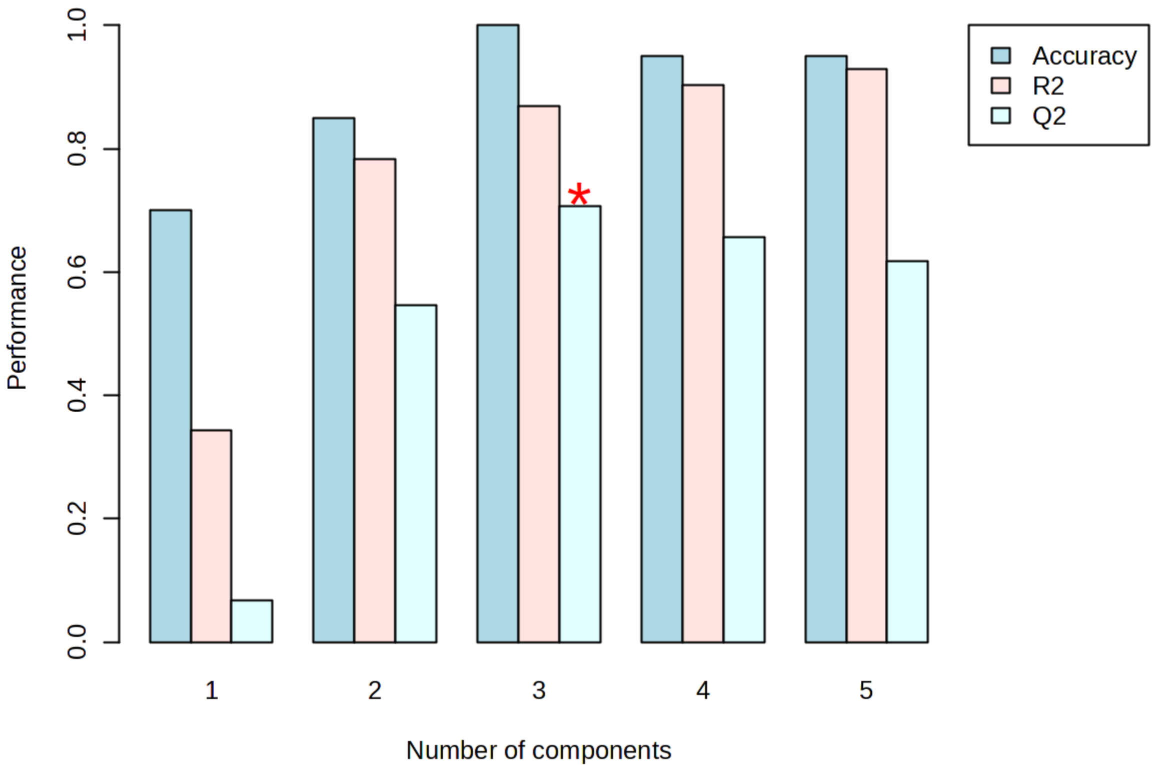

Sup. I Fig. 2: Cross-validation of PLS-DA analysis from MetaboAnalyst. Q2 is an estimate of the predictive ability of the model. The Q2 value indicates that the model in Fig. 1 is predictive. (Szymańska E., et al., Metabolomics. 2012, 8, 3-16, doi: 10.1007/s11306-011-0330-3)

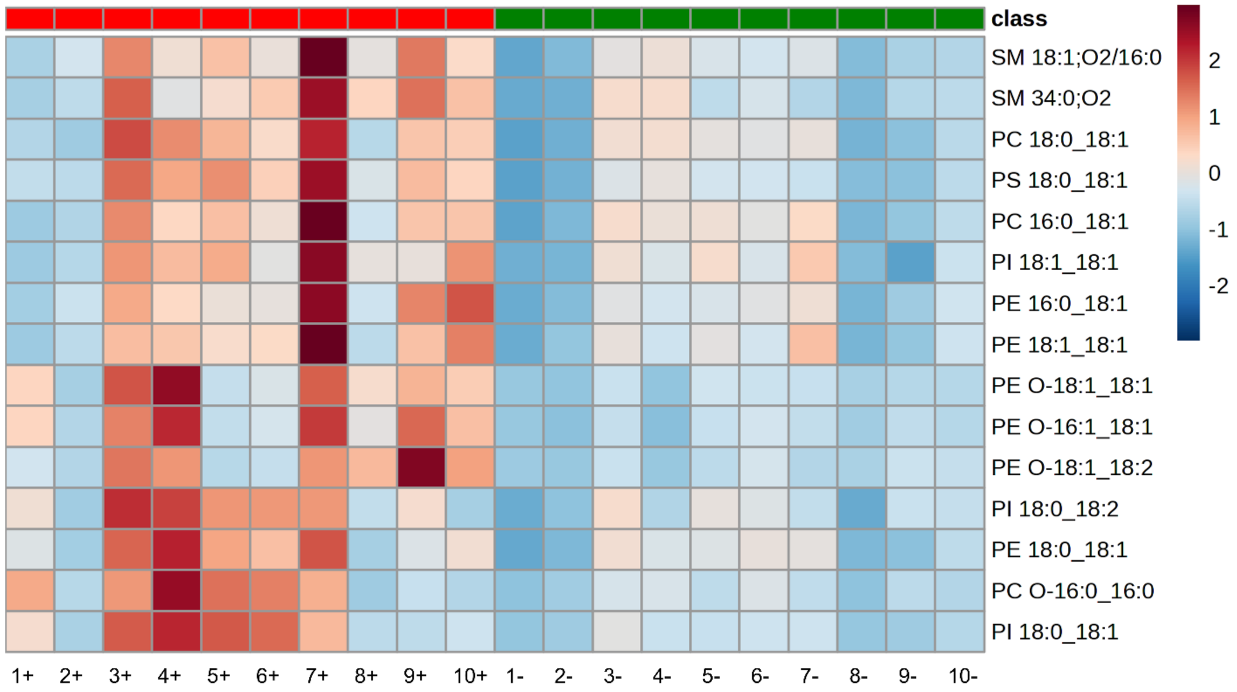

Sup. I Fig. 3: Heatmap of top 15 lipid features based on paired t-test (p-value < 0.05); Red – PI-ATM, Green – AI-ATM; the numerals presented below the columns correspond to individual sample/donor identifiers; with "+" and "-" designating samples of PI-ATM and NI-ATM origin, respectively.

Sup. I Tab. 1: Upregulated lipids in inflammatory macrophages identified by volcano plot (fold change > 2 and p-value < 0.05 by Student's t-test, FDR adjusted).

**Lipid**	**FC**	**Log_2(FC)**	**p-value (FDR)**	**-log_{10}(p)**
PI 18:1_18:1	2.6567	1.4096	0.013057	1.8842
PS 18:0_18:1	2.3833	1.2529	0.013057	1.8842
PE 18:0_18:1	2.1842	1.1271	0.013057	1.8842
PC 18:0_18:1	2.0181	1.013	0.013057	1.8842
PE 18:1_18:1	2.0823	1.0582	0.027665	1.5581
PE O-16:1_18:1	5.7736	2.5295	0.029808	1.5257
PI 18:0_18:1	4.6979	2.232	0.029808	1.5257
PI 18:0_18:2	3.0475	1.6076	0.029808	1.5257
SM 34:0;O2	2.3403	1.2267	0.029808	1.5257
PE 16:0_18:1	2.1888	1.1302	0.029808	1.5257
SM 18:1;O2/16:0	2.075	1.0531	0.029808	1.5257
PC 16:0_18:1	1.841	0.88048	0.029808	1.5257
PE O-18:1_18:1	7.7109	2.9469	0.03347	1.4753
PE O-18:1_18:2	6.6839	2.7407	0.03347	1.4753
PC O-16:0_16:0	3.17	1.6645	0.03347	1.4753
PC O-16:2_18:0	5.5509	2.4727	0.04199	1.3769
PC O-16:0_18:1	2.3329	1.2221	0.04199	1.3769
SM 32:1;O2	1.9328	0.95067	0.04199	1.3769
LPS 15:0	1.6725	0.742	0.04199	1.3769
