# Supplementary material for: Lipidomics of polarized macrophages in the human adipose tissue
Source: Sci Rep. 2025 Dec 19;16:3018. doi: 10.1038/s41598-025-32912-z (PMC12827255; doi:10.1038/s41598-025-32912-z)
Supplement: Supplementary file 2 — Supplementary Material 2 [file 41598_2025_32912_MOESM2_ESM.pdf]

Supplemental data II.
Lipidomics of polarized macrophages in the human adipose tissue

Vladimír Vrkoslav[a], Kateřina Pražáková[a,b], Štěpán Strnad[a], Karel Paukner [c,d], Barbora Muffová[c,d], Soňa Kauerová[c], Jiří Froněk[c], David Sýkora[b], Josef Cvačka[a,e], Rudolf Poledne[c], Marek Petráš[g], Ivana Králová Lesná[c,f*]

[a] *Institute of Organic Chemistry and Biochemistry of the Czech Academy of Sciences, Prague, Czech Republic*

[b] *Department of Analytical Chemistry, University of Chemistry and Technology Prague, Prague, Czech Republic*

[c] *Institute for Clinical and Experimental Medicine, Prague, Czech Republic*

[d] *Department of Physiology, Faculty of Science, Charles University in Prague, Prague, Czech Republic*

[e] *Department of Analytical Chemistry, Faculty of Science, Charles University in Prague, Prague, Czech Republic*

[f] *Department of Anesthesiology, Resuscitation and Intensive Care Medicine, 1st Faculty of Medicine, Charles University and Military University Hospital, Czech Republic*

[g] *Department of Epidemiology and Biostatistics, Charles University in Prague-Third Faculty of Medicine Charles University, 100 00 Prague, Czech Republic*

Sup. II Tab. 1: List of all annotated lipids in the adipose-tissue macrophage lipidome*.

					m (ng/100 000 macrophages)																			
Lipid	Class	RT (min)	Adduct type	Average mz	-1	1	-2	2	-3	3	-4	4	-5	5	-6	6	-7	7	-8	8	-9	9	-10	10
Cer 18:1;O2/16:0	Cer_NS	11.05	[M+H-H2O]+	520.50848	0.17	0.36	0.38	0.38	1.34	1.31	2.18	0.95	1.46	1.01	0.85	0.78	1.14	2.03	0.33	0.51	0.74	0.67	0.47	0.83
Cer 18:1;O2/18:0	Cer_NS	12.567	[M+H-H2O]+	548.54041	0.05	0.05	0.12	0.15	0.27	0.24	0.79	0.15	0.29	0.20	0.20	0.19	0.22	0.40	0.12	0.12	0.25	0.25	0.14	0.31
Cer 18:1;O2/20:0	Cer_NS	14.272	[M+H-H2O]+	576.57111	0.07	0.06	0.14	0.17	0.27	0.22	0.86	0.12	0.27	0.22	0.17	0.16	0.28	0.47	0.13	0.13	0.26	0.30	0.14	0.27
Cer 18:1;O2/22:0	Cer_NS	16.008	[M+H-H2O]+	604.60236	0.20	0.19	0.44	0.49	1.01	0.81	2.02	0.46	0.89	0.77	0.60	0.50	0.96	1.61	0.41	0.42	0.78	0.78	0.49	0.89
Cer 18:1;O2/23:0	Cer_NS	16.763	[M+H-H2O]+	618.61835	0.18	0.12	0.36	0.36	0.52	0.39	1.15	0.22	0.64	0.42	0.33	0.35	0.63	1.00	0.27	0.29	0.55	0.54	0.30	0.64
Cer 18:1;O2/24:0	Cer_NS	17.43	[M+H-H2O]+	632.63458	0.41	0.46	0.83	0.91	1.67	1.50	2.86	0.89	1.57	1.32	0.93	1.21	1.47	2.48	0.74	0.68	1.40	1.47	0.81	1.62
Cer 18:1;O2/24:1	Cer_NS	15.844	[M+H-H2O]+	630.61871	0.19	0.41	0.36	0.41	1.46	1.43	1.73	1.33	1.26	1.00	0.87	0.85	1.39	1.68	0.41	0.46	0.59	0.55	0.52	0.75
Cer 18:1;O2/26:0	Cer_NS	18.532	[M+H-H2O]+	660.66504	0.26	0.19	0.67	0.60	0.48	0.38	1.08	0.34	0.86	0.75	0.22	0.62	0.44	1.09	0.39	0.42	0.92	0.85	0.36	0.85
Cer 18:2;O2/24:0	Cer_NS	16.08	[M+H-H2O]+	630.61859	0.07	0.09	0.12	0.19	0.39	0.43	0.65	0.20	0.43	0.41	0.34	0.23	0.52	0.94	0.13	0.19	0.17	0.38	0.21	0.38
Cer 18:2;O2/24:1	Cer_NS	14.215	[M+H-H2O]+	628.6026	0.03	0.11	0.12	0.16	0.39	0.34	0.48	0.33	0.42	0.35	0.25	0.21	0.47	0.66	0.10	0.16	0.11	0.25	0.17	0.27
Cer 20:0;O2/24:0	Cer_NDS	18.868	[M+H]+	680.69208	0.37	0.30	1.03	0.92	0.57	0.48	1.06	0.48	1.00	1.04	0.28	0.88	0.74	1.59	0.60	0.63	1.46	1.27	0.74	1.27
Cer 20:1;O2/26:0	Cer_NS	19.417	[M+H-H2O]+	688.69623	0.27	0.18	0.77	0.60	0.48	0.35	0.77	0.31	0.86	0.70	0.20	0.59	0.43	1.11	0.35	0.39	0.85	1.02	0.32	0.78
Cer 20:1;O2/28:0	Cer_NS	20.145	[M+H-H2O]+	716.72827	0.36	0.20	0.88	0.78	0.51	0.36	0.62	0.33	0.94	0.87	0.20	0.65	0.46	1.26	0.42	0.47	0.90	1.30	0.36	0.92
Cer 22:1;O2/28:0	Cer_NS	20.761	[M+H-H2O]+	744.75873	0.35	0.17	0.79	0.74	0.54	0.33	0.50	0.32	0.75	0.77	0.19	0.60	0.42	1.28	0.37	0.42	0.83	1.28	0.27	0.81
Cer 24:0;O2/24:0	Cer_NDS	20.368	[M+H]+	736.75397	0.57	0.37	1.43	1.32	0.86	0.77	0.94	0.73	1.53	1.77	0.42	1.35	1.00	2.73	0.76	0.84	1.56	2.13	0.64	1.57
CL 18:1_18:2_18:2_18:2	CL	19.664	[M-H]-	1449.98364	0.20	0.78	0.05	0.52	2.75	5.76	0.53	1.92	2.39	0.67	2.98	1.54	2.50	4.58	0.10	0.80	0.23	4.10	1.63	2.23
CL 18:2_18:2_18:2_18:2	CL	19.145	[M-H]-	1447.96704	1.23	1.94	1.52	2.79	6.89	15.54	1.38	4.59	8.18	4.86	7.96	3.64	7.30	14.29	2.59	4.59	3.91	14.97	4.57	5.39
FA 16:0	FA	6.652	[M-H]-	255.23306	1.03	0.40	0.38	0.99	0.34	0.63	0.39	0.62	0.83	0.96	0.35	0.83	0.63	1.95	0.44	0.41	1.19	2.32	0.45	1.08
FA 18:0	FA	7.464	[M-H]-	283.2645	1.00	0.33	0.39	0.95	0.33	0.61	0.36	0.56	0.79	1.10	0.35	0.86	0.64	1.98	0.49	0.51	1.10	2.21	0.58	0.96
LPC 15:0	LPC	3.019	[M+H]+	482.3237	1.41	0.61	1.35	1.80	0.85	1.60	2.08	0.79	1.69	3.44	0.84	2.23	1.37	5.60	2.25	1.34	4.07	7.64	1.31	2.41
LPC 16:0	LPC	4.145	[M+H]+	496.33942	0.24	0.19	0.42	0.69	0.79	1.06	2.64	0.55	1.48	1.42	0.79	1.43	0.95	2.43	0.31	0.44	0.74	1.71	0.85	0.70
LPC 20:4	LPC	3.175	[M+H]+	544.33942	0.03	0.01	0.32	0.54	0.22	0.30	0.86	0.08	0.30	0.93	0.24	0.49	0.29	0.71	0.16	0.11	0.10	0.34	0.47	0.10
LPE 15:0	LPE	2.952	[M+H]+	440.27689	4.21	1.52	3.65	3.48	2.67	4.75	4.37	2.36	4.14	6.74	2.77	5.68	3.33	18.61	8.26	2.75	15.81	11.59	2.68	5.66
LPE 18:1	LPE	4.679	[M+H]+	480.30826	0.33	0.27	0.91	1.88	0.58	1.80	3.55	2.65	0.48	10.09	0.88	13.05	0.79	3.37	0.15	0.37	0.25	0.68	0.95	0.96
LPE 18:2	LPE	3.135	[M+H]+	478.29199	0.57	0.36	1.25	2.84	0.52	1.78	1.67	1.21	0.80	5.36	0.86	6.66	0.67	3.28	0.79	0.83	1.24	2.90	1.23	1.37
LPE 20:4	LPE	3.111	[M+H]+	502.29248	0.11	0.34	3.09	4.52	1.88	3.40	9.21	2.45	1.83	15.32	2.49	13.35	1.87	8.14	0.97	0.63	0.86	2.19	3.57	1.27
LPE 22:5	LPE	3.209	[M+H]+	528.30798	0.00	0.00	0.52	0.80	0.26	0.19	0.72	0.07	0.35	2.05	0.18	0.76	0.32	0.29	0.00	0.02	0.00	0.18	0.30	0.04
LPS 15:0	LPS	2.052	[M-H]-	482.25256	1.17	0.68	0.70	0.70	0.52	1.63	0.35	0.68	1.52	2.67	0.93	1.40	1.82	3.63	1.10	1.73	3.66	4.66	0.45	2.06
PC 16:0_16:0	PC	10.926	[M+HCOO]-	778.56146	4.95	10.69	10.62	18.88	43.07	51.47	40.93	23.89	35.85	39.43	36.63	26.48	36.05	83.73	10.04	21.08	16.92	40.39	23.68	36.25
PC 16:0_18:0	PC	12.374	[M+HCOO]-	806.59283	1.15	2.30	1.95	3.30	8.58	11.40	8.86	5.91	6.28	7.59	6.47	5.24	6.07	13.62	1.81	4.99	2.04	5.83	4.19	6.15
PC 16:0_18:1	PC	10.98	[M+HCOO]-	804.57678	10.82	31.13	20.60	38.43	70.38	106.74	64.22	74.76	65.64	86.10	59.45	66.01	73.15	167.24	19.97	49.24	28.04	83.27	43.02	83.28
PC 16:0_18:2	PC	9.943	[M+HCOO]-	802.56165	6.10	9.74	10.05	28.34	37.87	67.91	19.15	21.75	33.09	40.86	34.01	29.23	29.17	85.06	11.09	37.54	21.02	91.87	26.93	57.01
PC 16:0_20:4	PC	9.709	[M+HCOO]-	826.5611	3.16	3.05	2.70	6.71	15.36	18.16	5.16	4.80	12.60	10.38	13.20	5.31	11.53	28.70	5.46	15.69	4.35	20.30	8.24	15.50
PC 18:0_18:1	PC	12.437	[M+HCOO]-	832.60889	3.49	13.22	5.74	10.89	22.83	42.75	23.13	35.47	20.39	30.37	19.74	24.07	21.10	47.12	6.45	13.89	8.53	28.13	14.04	26.50
PC 18:0_18:2	PC	11.227	[M+HCOO]-	830.59253	6.11	9.20	10.07	23.23	42.53	58.79	23.63	21.10	34.61	34.72	38.32	25.05	30.09	65.68	12.46	28.54	15.98	64.63	28.60	45.38
PC 18:0_20:3	PC	11.56	[M+HCOO]-	856.60828	1.12	1.02	0.87	2.11	4.98	6.11	3.15	2.35	4.66	3.56	4.98	2.09	4.09	8.27	1.39	2.90	1.20	5.38	3.33	4.65
PC 18:0_20:4	PC	10.958	[M+HCOO]-	854.5932	4.82	3.86	4.22	8.66	28.03	30.88	10.03	8.17	20.80	16.17	22.45	8.06	19.70	44.46	10.13	20.52	7.35	25.62	13.88	22.74
PC 18:0_22:5	PC	10.935	[M+HCOO]-	880.60852	0.17	0.14	0.15	0.37	2.68	1.95	0.77	0.56	2.18	1.01	1.87	0.59	2.28	4.25	0.50	1.13	0.08	0.90	0.97	1.15
PC 18:1_18:1	PC	11.031	[M+HCOO]-	830.59296	3.54	9.84	6.22	7.77	23.42	28.78	19.14	25.63	26.73	20.84	15.74	18.75	28.35	46.03	5.73	9.67	6.00	11.56	12.13	17.93
PC O-16:0_16:0	EtherPC	11.806	[M+HCOO]-	764.58252	0.26	7.71	0.99	1.87	3.20	8.48	3.34	14.08	2.19	9.90	3.53	9.34	2.23	7.51	0.26	0.90	2.06	2.52	1.73	1.68
PC O-16:0_18:1	EtherPC	11.828	[M+HCOO]-	790.5979	0.66	8.58	1.60	2.53	5.76	12.81	5.05	20.66	4.06	12.43	6.66	13.79	4.60	11.21	1.07	1.69	3.37	3.53	3.21	3.23
PC O-16:0_20:4	EtherPC	10.442	[M+HCOO]-	812.58203	0.56	2.42	0.62	0.84	4.84	4.41	1.29	3.76	3.19	2.30	4.51	2.21	2.76	5.05	0.37	2.09	0.99	3.14	2.37	1.86
PC O-16:1_16:0	EtherPC	11.595	[M+HCOO]-	762.56622	0.57	1.80	0.00	0.73	3.55	5.41	0.06	2.64	3.37	1.05	2.58	0.83	3.33	7.15	0.86	1.93	0.94	3.37	1.29	2.00
PC O-16:1_20:4	EtherPC	10.198	[M+HCOO]-	810.56635	0.96	1.21	0.00	0.97	6.41	6.26	0.05	1.59	6.95	0.14	3.46	0.06	5.65	10.63	1.78	5.00	0.74	4.84	1.51	2.42
PC O-16:2_18:0	EtherPC	11.64	[M+HCOO]-	788.58203	0.06	1.48	0.03	0.72	1.50	5.14	0.02	3.01	1.25	0.56	0.99	0.73	1.52	6.27	0.36	1.97	1.06	4.98	0.62	2.07
PC O-18:0_20:4	EtherPC	11.806	[M+HCOO]-	840.61395	0.06	1.92	0.07	0.17	1.86	2.22	0.40	3.52	1.07	1.52	1.59	1.65	0.83	1.40	0.21	0.55	0.15	0.23	0.69	0.35
PC O-18:1_20:4	EtherPC	10.443	[M+HCOO]-	838.5976	0.38	2.12	0.68	1.26	5.06	4.38	0.78	2.88	3.21	2.39	4.07	1.44	3.52	5.62	0.55	1.76	1.04	1.94	2.11	1.60
PE 16:0_18:1	PE	11.353	[M-H]-	716.52429	0.58	1.77	1.08	2.78	3.45	5.94	3.02	4.54	3.17	3.85	3.46	3.67	3.97	10.08	0.83	2.85	1.70	6.84	2.89	7.97
PE 16:0_18:2	PE	10.264	[M-H]-	714.50842	0.10	0.20	0.26	0.76	2.03	3.12	0.82	1.23	1.64	0.89	2.54	1.69	1.61	4.04	0.04	0.75	1.27	4.70	1.22	3.46
PE 18:0_18:1	PE	12.88	[M-H]-	744.55621	1.66	10.59	3.18	5.83	12.93	23.64	10.34	28.34	10.70	19.10	11.82	16.78	11.48	24.85	3.21	5.87	4.09	10.49	7.84	12.81
PE 18:0_18:2	PE	11.61	[M-H]-	742.54016	1.45	2.73	2.56	5.15	9.73	11.34	5.12	6.59	7.92	6.20	10.23	6.35	7.13	13.48	2.39	5.29	4.28	13.99	7.02	10.16
PE 18:0_20:3	PE	11.949	[M-H]-	768.55499	0.28	0.80	0.48	1.34	2.23	2.40	1.20	1.27	2.14	1.42	2.67	1.19	2.45	3.56	0.52	1.09	0.64	3.28	1.74	2.54
PE 18:0_20:4	PE	11.317	[M-H]-	766.53992	5.24	5.15	4.89	8.93	27.53	22.25	8.01	7.89	20.03	9.36	25.11	8.49	19.13	28.47	8.00	11.27	8.01	23.27	15.08	21.92
PE 18:0_22:4	PE	12.348	[M-H]-	794.57159	0.36	0.69	0.32	0.67	2.22	2.27	0.83	1.33	1.23	1.10	2.15	0.89	1.86	2.70	0.30	1.08	0.42	1.54	1.15	1.66
PE 18:0_22:5	PE	11.296	[M-H]-	792.55579	0.43	0.27	0.36	0.35	3.10	1.97	0.50	0.55	2.33	0.65	2.31	0.49	2.23	2.84	0.52	0.88	0.61	1.43	1.54	1.36
PE 18:0_22:6	PE	10.863	[M-H]-	790.54065	0.27	0.06	0.26	0.23	2.73	1.56	0.20	0.27	3.15	0.77	1.77	0.25	1.88	2.25	0.46	0.70	0.27	1.07	1.76	1.86
PE 18:1_18:1	PE	11.402	[M-H]-	742.54022	0.42	1.37	1.15	2.05	3.27	4.69	2.48	4.42	3.11	3.76	2.63	3.90	4.65	9.52	0.70	2.07	1.19	4.59	2.45	6.05
PE 18:1_20:4	PE	10.054	[M-H]-	764.5246	0.65	0.69	0.51	1.08	3.57	2.99	1.03	1.13	3.49	1.59	3.98	1.63	3.46	5.32	0.80	1.50	1.37	3.74	2.25	3.94
PE O-16:1_18:1	EtherPE	12.105	[M-H]-	700.52954	1.00	10.11	0.24	2.91	4.20	16.63	0.10	22.48	4.45	4.12	5.42	5.56	4.06	21.30	1.59	6.97	3.97	18.38	3.11	12.08
PE O-16:1_18:2	EtherPE	10.906	[M-H]-	698.51367	0.38	2.07	0.03	3.09	2.63	9.42	0.00	5.64	1.99	1.21	4.66	2.07	1.68	10.65	0.95	6.48	3.51	23.15	2.73	9.20
PE O-16:1_20:4	EtherPE	10.595	[M-H]-	722.51337	3.74	8.86	0.48	5.07	19.60	23.13	0.84	13.49	16.88	3.01	16.53	3.62	14.55	43.17	5.67	14.80	7.34	28.64	8.75	20.03
PE O-16:1_22:4	EtherPE	11.579	[M-H]-	750.54486	2.04	3.29	0.24	1.80	7.28	8.24	0.21	5.06	6.48	1.19	7.65	1.22	6.48	15.76	2.55	5.51	3.54	9.86	2.79	6.18
PE O-16:1_22:5	EtherPE	10.606	[M-H]-	748.52936	1.89	4.65	0.08	2.08	11.60	12.82	0.14	7.21	11.38	1.89	9.67	1.84	9.83	26.87	3.02	9.12	3.59	13.43	4.95	11.39
PE O-16:1_22:6	EtherPE	10.161	[M-H]-	746.5141	0.54	0.82	0.03	0.37	5.08	6.13	0.00	1.16	6.97	0.85	3.25	0.22	3.22	9.70	0.66	2.95	1.18	5.74	2.22	5.14
PE O-18:1_18:1	EtherPE	13.718	[M-H]-	728.56128	0.38	8.09	0.03	1.28	3.40	16.12	0.07	21.14	3.80	3.21	3.60	4.75	3.33	15.57	1.36	7.11	2.22	10.61	2.22	8.78
PE O-18:1_18:2	EtherPE	12.364	[M-H]-	726.54523	0.45	4.48	0.20	2.10	3.81	17.15	0.00	14.91	2.78	2.40	4.80	3.49	2.12	14.97	1.50	12.00	3.95	26.66	3.50	14.13
PE O-18:1_20:4	EtherPE	12.012	[M-H]-	750.54541	3.69	19.42	0.48	3.81	24.29	43.89	1.06	34.03	18.17	7.03	17.86	6.92	14.52	58.37	8.28	31.53	5.99	33.04	9.50	25.46
PE O-18:1_22:4	EtherPE	13.118	[M-H]-	778.57642	0.62	2.52	0.00	0.46	4.09	5.58	0.00	4.57	2.74	0.47	3.63	0.90	2.55	5.46	1.48	3.52	0.86	3.11	1.36	2.36
PE O-18:1_22:5	EtherPE	11.985	[M-H]-	776.56122	0.71	2.68	0.00	0.53	6.61	7.98	0.12	5.06	5.75	1.03	4.24	1.03	3.69	8.89	1.72	3.32	0.99	3.71	1.95	3.19
PE O-18:1_22:6	EtherPE	11.497	[M-H]-	774.5451	0.47	1.10	0.00	0.05	3.61	5.03	0.09	1.35	3.94	1.03	1.81	0.24	1.66	6.21	0.90	2.89	0.46	3.08	1.43	3.32
PI 16:0_20:4	PI	8.921	[M-H]-	857.51929	0.31	0.64	0.50	1.13	2.61	2.94	0.67	1.26	2.17	1.34	2.25	1.18	2.10	4.08	0.70	1.68	0.93	3.21	1.44	2.51
PI 18:0_18:1	PI	11.277	[M-H]-	863.56665	0.04	2.19	0.25	0.48	1.67	4.94	1.06	5.84	1.04	4.97	1.14	4.68	1.15	3.17	0.09	0.84	0.27	0.88	0.70	1.16
PI 18:0_18:2	PI	10.203	[M-H]-	861.55042	0.07	1.56	0.39	0.58	1.66	3.59	0.73	3.40	1.42	2.58	1.26	2.57	0.93	2.57	0.05	0.93	1.00	1.64	0.96	0.62
PI 18:0_20:3	PI	10.522	[M-H]-	887.56653	0.19	2.01	0.48	0.64	2.23	4.90	1.07	7.17	2.23	2.89	2.56	4.31	2.41	4.03	0.36	0.60	0.84	1.35	1.31	1.14
PI 18:0_20:4	PI	10	[M-H]-	885.55157	6.87	16.48	8.40	18.95	42.68	60.82	12.21	35.70	38.55	34.79	35.46	28.27	35.45	74.99	12.29	27.19	14.01	51.32	21.80	32.54
PI 18:1_18:1	PI	10.04	[M-H]-	861.55084	0.27	0.71	0.35	0.99	1.81	2.93	1.45	2.48	1.93	2.68	1.42	1.59	2.29	4.64	0.44	1.67	0.00	1.70	1.26	2.96
PI 18:1_20:4	PI	8.965	[M-H]-	883.53546	0.90	1.28	1.16	2.23	5.49	5.80	1.31	2.62	5.67	5.28	4.91	2.75	6.09	13.76	1.57	3.95	1.65	6.31	2.89	5.49
PS 18:0_18:1	PS	11.48	[M-H]-	788.54584	7.64	36.03	13.93	34.15	44.70	94.10	48.91	76.80	41.01	83.94	40.18	62.48	37.65	121.28	18.58	44.09	19.75	69.81	33.72	60.66
SM 18:1;O2/16:0	SM	9.683	[M+H]+	703.57458	10.95	33.38	19.66	49.01	57.15	103.24	61.89	63.74	51.68	81.87	48.66	60.31	53.19	163.17	20.37	57.94	33.22	108.36	36.97	69.24
SM 24:0;O2/14:1	SM	12.518	[M+H]+	759.63824	0.54	0.56	1.26	3.40	2.79	4.53	4.01	1.29	2.49	2.72	2.60	1.45	2.58	7.35	1.15	3.29	1.23	5.57	2.43	4.93
SM 24:2;O2/10:0	SM	8.679	[M+H]+	701.55908	0.26	0.49	0.65	1.50	1.14	1.76	1.83	1.14	1.69	2.19	1.08	0.99	1.44	4.80	0.73	1.97	0.76	2.85	1.12	2.35
SM 32:1;O2	SM	8.576	[M+H]+	675.54346	0.28	0.65	0.58	1.29	0.96	1.68	1.57	1.38	1.40	2.35	1.11	1.34	1.20	3.85	0.53	1.25	0.68	2.01	0.89	1.76
SM 33:1;O2	SM	9.114	[M+H]+	689.5592	0.18	0.39	0.45	1.10	0.72	1.21	1.24	0.88	1.20	1.70	0.92	0.89	0.98	3.16	0.31	0.86	0.41	1.74	0.62	1.04
SM 34:0;O2	SM	10.185	[M+H]+	705.59027	0.20	0.66	0.28	0.88	1.47	2.61	1.46	1.20	0.88	1.44	1.09	1.70	0.77	3.30	0.36	1.59	0.78	2.49	0.84	1.79
SM 35:1;O2	SM	10.35	[M+H]+	717.59155	0.04	0.19	0.05	0.15	0.50	0.76	0.75	0.49	0.43	0.36	0.57	0.49	0.39	1.46	0.07	0.48	0.00	0.66	0.33	0.15
SM 36:1;O2	SM	11.015	[M+H]+	731.60657	0.59	0.90	1.44	3.90	3.24	5.76	4.61	1.87	2.41	2.57	3.39	2.12	2.36	5.92	1.62	4.09	1.19	6.18	2.61	4.97
SM 38:2;O2	SM	11.102	[M+H]+	757.62262	0.06	0.06	0.25	0.70	0.41	0.66	0.65	0.22	0.44	0.46	0.48	0.24	0.46	1.66	0.13	0.48	0.19	1.24	0.47	1.00
SM 39:1;O2	SM	13.303	[M+H]+	773.65381	0.14	0.12	0.34	0.83	0.44	0.73	0.85	0.27	0.71	0.85	0.58	0.38	0.63	1.89	0.25	0.59	0.23	1.11	0.49	0.91
SM 40:1;O2	SM	14.176	[M+H]+	787.66895	1.64	1.73	2.77	7.02	7.27	11.18	9.03	3.25	6.13	7.06	5.43	3.84	6.20	16.80	2.98	9.01	3.09	12.79	6.19	11.20
SM 40:2;O2	SM	12.347	[M+H]+	785.65381	0.07	0.22	0.21	0.47	0.37	0.56	0.48	0.48	0.56	0.80	0.46	0.36	0.47	1.39	0.18	0.47	0.22	0.65	0.37	0.73
SM 40:9;O2	SM	7.638	[M+H]+	771.55072	0.14	0.01	0.87	0.98	0.01	0.05	1.54	0.02	0.02	1.24	0.05	0.77	0.01	0.15	0.07	0.02	0.29	0.19	0.24	0.38
SM 41:1;O2	SM	15.049	[M+H]+	801.68445	0.66	0.53	1.09	2.43	2.01	2.95	3.15	0.92	2.59	2.30	1.95	1.24	2.63	5.95	1.17	2.48	1.00	3.82	1.75	2.68
SM 41:2;O2	SM	13.153	[M+H]+	799.6698	0.10	0.14	0.22	0.49	0.33	0.48	0.54	0.35	0.64	0.54	0.47	0.30	0.49	1.08	0.17	0.38	0.16	0.66	0.32	0.53
SM 42:1;O2	SM	15.898	[M+H]+	815.70062	1.42	1.84	2.02	4.20	6.79	9.04	8.16	3.29	5.16	5.10	4.84	3.48	5.47	11.20	2.40	5.28	2.93	7.90	5.12	7.78
SM 42:8;O2	SM	8.306	[M+H]+	801.59772	0.67	0.40	1.74	2.29	0.77	1.47	4.79	1.00	1.65	4.94	1.36	2.35	1.75	4.59	1.70	1.16	2.07	3.23	0.55	2.93
ST 27:1;O	ST	7.964	[M+H-H2O]+	369.3479	159.17	128.47	180.70	229.58	270.54	487.18	357.86	374.88	458.30	1363.15	192.67	756.64	397.17	1092.11	279.10	216.86	391.26	322.74	104.81	420.24

*The abbreviations follow the MS-DIAL system (https://systemsomicslab.github.io/compms/msdial/lipidnomenclature.html)
